# Schlafen Family Intra-Regulation by IFN-α2 in Triple-Negative Breast Cancer

**DOI:** 10.3390/cancers15235658

**Published:** 2023-11-30

**Authors:** Savannah R. Brown, Emilie E. Vomhof-DeKrey, Sarmad Al-Marsoummi, Nicholas D. Brown, Kole Hermanson, Marc D. Basson

**Affiliations:** 1 Department of Pathology, School of Medicine and the Health Sciences, University of North Dakota, Grand Forks, ND 58202, USA; savannah.corradi@und.edu (S.R.B.); emilie.dekrey@und.edu (E.E.V.-D.); nicholas.d.brown@und.edu (N.D.B.); 2 Department of Surgery, School of Medicine and the Health Sciences, University of North Dakota, Grand Forks, ND 58202, USA; kole.hermanson@und.edu; 3 Department of Biomedical Sciences, School of Medicine and the Health Sciences, University of North Dakota, Grand Forks, ND 58202, USA; 4 Department of Surgery, Northeast Ohio Medical University, Rootstown, OH 44272, USA; 5 Department of Anatomy and Neurobiology, Northeast Ohio Medical University, Rootstown, OH 44272, USA

**Keywords:** Schlafen 12, TNBC, IFN-𝛼2, AdvShSLFN12, cell viability

## Abstract

**Simple Summary:**

TNBC is an aggressive subtype of breast cancer that responds differently to treatment. SLFN12 expression correlates with better survival in TNBC patients, but there is no known treatment to upregulate SLFN12. This study indicates that IFN-α2 treatment induces SLFN5, SLFN11, SLFN12, SLFN12-Like, SLFN13, and SLFN14 expression in TNBC while simultaneously reducing cell viability. However, IFN-α2 does not work through SLFN12 exclusively but rather through the SLFN family as a whole. Following simultaneous SLFN knockdown, IFN-α2 signaling initiates a complex signaling cascade among Schlafen family members in TNBC.

**Abstract:**

Triple-negative breast cancer (TNBC) has a poor prognosis and no targeted therapy for treatment. The Schlafen gene family, particularly SLFN12, critically mediates TNBC biology. Higher expression of SLFN12 correlates with decreased TNBC viability and increased chemosensitivity and patient survival, yet no treatment is known to upregulate SLFN12 in TNBC. We hypothesized that Interferon-α (IFN-α2) upregulates SLFN12 in TNBC, subsequently reducing cell viability. We utilized short hairpin adenovirus to knockout SLFN12 (AdvShSLFN12) in MDA-MB-231, Hs-578T, and BT-549 TNBC cells. Cells were treated with AdvShSLFN12 and IFN-α2. After treatment, TNBC cell viability, SLFN family mRNA, and protein expression were analyzed. Treating TNBC cells with IFN-α2 increased SLFN12 expression and reduced cell viability. However, when AdvShSLFN12 knocked down SLFN12 during IFN-α2 treatment, TNBC cell viability was still reduced. We, therefore, investigated the potential involvement of other SLFN members IFN-α2 effects on cell viability. IFN-α2 increased SLFN5, SLFN12-Like, and SLFN14 but not SLFN11 or SLFN13. During AdvShSLFN12 + IFN-α2 treatment, the expressions of SLFN5, SLFN12-Like, and SLFN14 further increased. However, when siRNA knocked down SLFN5, SLFN12-Like, and SLFN14, the IFN-α2 reduction in viability was blunted. Although the interpretation of these results may be limited by the potential interactions between different siRNAs, these data suggest a complex regulatory signaling cascade among SLFN family members. Targeting this cascade to manipulate SLFN levels may, in the future, offer the potential to manipulate the chemosensitivity of TNBC tumors.

## 1. Introduction

Breast cancer is the second most diagnosed cancer for women in the United States, and despite advances in cancer therapies, breast cancer death rates continue to increase by 0.5% each year [1]. In 2022, 288,000 women were diagnosed with breast cancer in the United States, and 43,000 died [1]. The treatment of breast cancer is a multifactorial process that assesses the stage, hormonal responses, gene mutations, growth rate, and age of the patient [2]. Triple-negative breast cancer (TNBC) is an aggressive basal-like subtype of breast cancer that lacks estrogen and progesterone receptors and human epidermal growth factor receptor 2 (HER2) [2,3]. Because TNBC lacks these receptors, receptor-targeted treatment for TNBC is not available. Only non-specific treatments, such as surgery, radiation, and chemotherapy, can be offered, even though they are less specific and may have harsh side effects [2,4]. The absence of hormonal receptors paired with an enriched CD44^+^CD24^−^ breast cancer stem cell (BCSC) population drives the chemoresistant, radioresistant, and aggressive nature of TNBC [2,3,5]. Therefore, the search for alternative treatment regimens for TNBC patients is critical. 

Recent advances have highlighted the diverse roles of the Schlafen (SLFN) family of proteins in cancer biology and their influence on patient survival [6]. Schlafens have a distinct molecular structure with minimal similarity to other proteins and are classified into three subgroups—small, intermediate, and long—based on structure and size [3,6,7]. Humans express intermediate Schlafens (SLFN12 and SLFN12-Like) and long Schlafens (SLFN5, SLFN11, SLFN13, and SLFN14) but lack the short Schlafens that are expressed in rodents [6,8]. The role of long SLFN proteins has been extensively studied in cancer, but less is known about the role of intermediate SLFNs in cancer. The intermediated SLFNs are not expected to act similarly to long SLFN proteins because long SLFNs are targeted to and act in the nucleus, while the intermediate SLFN12 and SLFN12L lack the nuclear targeting sequence [6,9]. Indeed, Slfn3, the closest rodent homolog to SLFN12, not only acts in the cytosol but is enhanced in activity if a nuclear exclusion sequence is added to its structure [10,11]. We have previously confirmed that SLFN12 reduces the aggressiveness of TNBC via post-transcriptional regulation of ZEB1 and that over-expression of SLFN12 sensitizes TNBC cells to chemotherapy drugs and radiation (Sarmad, Adahm). While understanding SLFN members is important, SLFN family interplay has not been examined, and there is no known method to upregulate SLFN family members in TNBC. In vitro, SLFN12 overexpression reduces TNBC cell viability, promotes tumor cell differentiation, and reduces tumor proliferation, perhaps leading to more favorable tumor biology [3,6,7]. In parallel, TNBC patients who express higher levels of SLFN12 have longer survival than patients whose tumors express lower levels of SLFN12 [3,7,12]. Furthermore, SLFN12 has been reported to sensitize TNBC to chemotherapy and radiation, indicating that SLFN12 levels may affect both the intrinsic tumor biology and the tumor’s response to treatment [7]. However, although SLFN12 levels may be important, we lack any drug therapy to upregulate SLFN12 in TNBC. Although SLFN proteins may play an important role in TNBC biology, there is no known method to manipulate them within TNBC cells. Furthermore, although understanding the effects of individual SLFN family members is important, the interplay among SLFN proteins is poorly understood and has not been examined.

Interferons, specifically IFN-α2, have been utilized as an adjuvant therapy for cancer treatment since the 1980s [12]. IFN-α2 treatment can be used as an immunotherapy to target lingering cancer cells to enhance suppressed T cell cytotoxicity [12,13]. Therefore, understanding the relationship between IFN-α2 and SLFN12 may provide insight into delivering targeted therapy for TNBC patients.

In this study, we hypothesized and demonstrated that IFN-α2 signaling induces SLFN12 overexpression along with a decrease in TNBC viability in three TNBC cell lines, MDA-MB-231, BT-549, and Hs-578T. However, when IFN-α2 induction of SLFN12 mRNA was blocked with AdvShSLFN12 treatment, TNBC cell viability was still decreased. These findings led us to explore the involvement of other SLFN members having a role in cell viability during IFN-𝛼2 signaling. 

## 2. Methods 

### 2.1. Cells and Reagents

Cell lines were obtained from the American Tissue Culture Collection (ATCC, Manassas, VA, USA). MDA-MB-231 cells and BT-549 cells were cultured in DMEM (Genesee Scientific, El Cajon, CA, USA), and Hs-578T cells were cultured in RPMI (Genesee Scientific). All cell lines were supplemented with 10% fetal bovine serum (FBS) (Genesee Scientific) Penicillin (10,000 units)/Streptomycin (10 mg/1 mL) (ThermoFisher Scientific, Waltham, MA, USA), and grown in 5% CO_2_ at 37 °C. Crystal violet C-6158 was from Sigma-Aldrich (Burlington, MA, USA). IFN-α2 was from Biolegend (San Diego, CA, USA). Primers are listed in Supplemental Table S1, and antibodies are listed in Supplemental Table S2.

### 2.2. Viral Constructs

Short hairpin RNA adenovector targeting SLFN12 was obtained from Vector Biolab (Malvern, PA, USA, #shADV-223642). The control virus was constructed from the pAdeno vector with only the CMV promoter, as described previously [8].

### 2.3. siRNA Studies

MDA-MB-231 cells were seeded into a six-well plate at 200,000 cells per well and allowed to attach for 24 h. Cells were treated for 48 h with control non-targeting siNT (40 pmol) (Dharmacon, Lafayette, CO, USA, D-001210–05-20), siSLFN5 (60 pmol), siSLFN12-Like (40 pmol), or siSLFN14 (40 pmol) (ThermoFisher Scientific) using Lipofectamine RNAiMAX (ThermoFisher Scientific, #13778150) transfection reagent in Opti-MEM medium. The lipofectamine RNAiMAX mixture (Mix A) and each siRNA mixture (Mix B) were made individually (Supplemental Table S3). Mixture A and mixture B were then combined in a 1:1 ratio and incubated for five minutes at room temperature. A total of 300 μL of siRNA mixtures were added into 2 mL of complete medium/well. Cells incubated for an additional 48 h were then harvested. Information on siRNA mixture concentrations is listed in Supplemental Tables S3 and S4.

### 2.4. qPCR

Denoted TNBC cells were seeded into 6-well plates at a density of 200,000 cells per well and allowed to attach for 24 h. Following 48 h of treatment with an experimental medium, RNA was isolated, and qPCR was performed, as previously described [7]. Expression was calculated from threshold cycle (Ct) values by 2^−ΔΔCt^ fold change using RPLP0 as the reference gene.

### 2.5. Cell Viability 

Cell viability was assessed utilizing a crystal violet-based assay. MDA, MB-231, BT-549, and Hs578T cells were seeded in six-well plates at 200,000 cells/well. After 24 h, cells were infected with adenoviral vectors at 4000 viral particles (VP)/cell, treated with IFN-α2 at 5500 units, or a combination of AdvshSLFN12 (4000 VP) and IFN-α2 (5500 units) for 48 h. Cells were *gently* washed with distilled water to remove the experimental medium and non-adherent cells. An amount of 150 μL of room temperature 0.50% crystal violet solution in methanol was added to each well for 30 min, followed by 100 rpm shaking to stain and fix adherent cells. Fixed cells were washed with distilled water three times and air-dried for 24 h. Cells were solubilized with 300 μL methanol per well and incubated at room temperature while shaking at 400 rpm; then, optical density was measured at 570 nm with the Tecan Spark^®^ Multimode Microplate Reader (Männedof, Switzerland). The optical density of the treated cells in each well was normalized to the mean of the control cells of the same cell type and multiplied by 100 to calculate the percentage of cell viability [7]. The crystal violet-based assay is an established technique we [7,8] and others [14,15] have previously utilized to determine the percentage of viable cells while excluding dead cells. Flow cytometry was utilized to evaluate the numbers and ratios of both viable and dead cells.

### 2.6. Flow Cytometry

MDA-MB-231 cells were stained for viability using the Zombie Aqua™ Fixable Viability Kit from Biolegend (San Diego, CA, USA), followed by using the FoxP3/Transcription factor fixation/permeabilization kit from eBioscience (San Diego, CA, USA). Intracellular staining was performed with the antibodies listed in Supplemental Table S2. Flow cytometry samples were acquired on a BD Symphony flow cytometer (BD, San Jose, CA, USA) and analyzed with FlowJo software Version 10.9.0 (TreeStar, Ashland, OR, USA) [3,16].

### 2.7. Statistical Analysis

Data are expressed as mean ± SEM and analyzed by GraphPad prism v9 (San Diego, CA, USA) using One-Way ANOVA followed by Šídák’s multiple comparisons test unless stated otherwise. All experiments were repeated a minimum of three times.

## 3. Results 

### 3.1. IFN-α2 Increases SLFN12 Expression and AdvShSLFN12 Prevents the IFN-α2 Associated Increase in SLFN12

**Figure 1:**
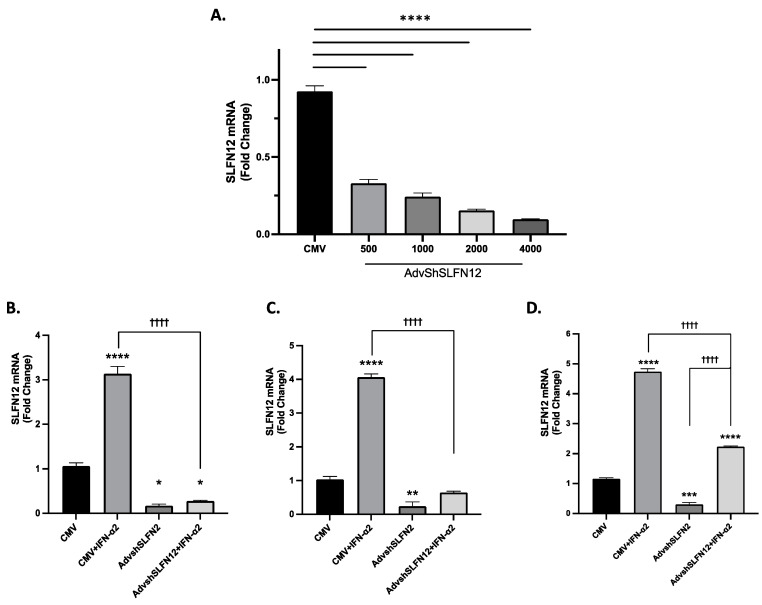
IFN-𝛼2 increases SLFN12 expression, whereas AdvShSLFN12 prevents the IFN-𝛼2-associated increase in SLFN12. (**A**) mRNA analysis by primer-probe RT-qPCR confirmed that the adenovirus short hairpin RNA for SLFN12 (AdvshSLFN12) is effective at knocking down SLFN12 in MDA-MB-231 cells after 48 h (*n* = 6, *p* < 0.0001). -qPCR analysis following treatment with IFN-𝛼2 was able to induce SLFN12 but not in the presence of AdvshSLFN12 after 48 h in (**B**) MDA-MB-231 (*n* = 6, *p* < 0.0001), (**C**) BT-549 (*n* = 3, *p* < 0.001), and (**D**) Hs-578T (*n* = 3, *p* < 0.0001). RPLP0 was used as a reference gene. All error bars shown represent the standard error of the mean. Asterisks denote significance between control and each condition, whereas crosses indicate significance between shown conditions. *p*-value is for both asterisks and crosses. * *p* < 0.05; ** *p* < 0.01; *** *p* < 0.001; **** *p* < 0.0001; †††† *p* < 0.0001.

To determine whether IFN-α2 was able to induce SLFN12 expression in TNBC, we utilized the well-defined and aggressive MDA-MB-231 cell line. We confirmed that AdvShSLFN12 was able to knock down SLFN12 expression (Figure 1A). Next, we demonstrated that IFN-α2 was able to induce SLFN12 expression alone, but when endogenous SLFN12 was knocked down with AdvShSLFN12, IFN-α2 was not able to induce SLFN12 expression due to the effectiveness of the AdvShSLFN12 in MDA-MB-231 cells (Figure 1B, Supplemental Figure S1). To confirm that this relationship was not idiosyncratic to one cell line, we performed the same experiment in BT-549 and Hs-578T TNBC cells. Once again, we observed that IFN-α2 was able to induce SLFN12 expression alone, but when SLFN12 is knocked out, IFN-α2 is no longer able to induce SLFN12 expression in BT-549 (Figure 1C, Supplemental Figure S2) or Hs578T (Figure 1D, Supplemental Figure S3) TNBC cells to the degree of IFN-α2 alone.

### 3.2. IFN-α2 Continues to Be Effective in Decreasing TNBC Cell Viability Despite a Knockdown in SLFN12

**Figure 2:**
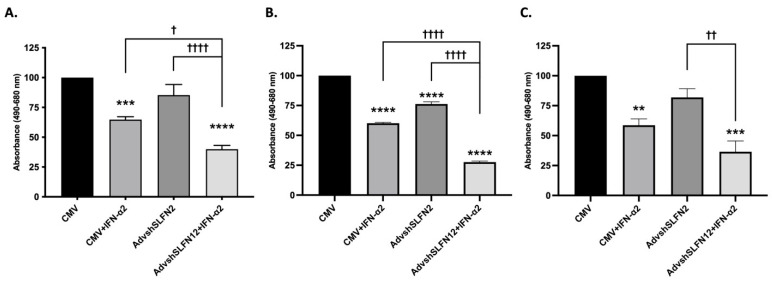
IFN-𝛼2 continues to be effective in decreasing TNBC cell viability despite a knockdown in SLFN12. Crystal violet assay showed a decrease in cell viability with IFN-α2 and a further decrease when AdvShSLFN12 and IFN-α2 were combined in (**A**) MDA-MB-231 (*n* = 6, *p* < 0.0001), (**B**) BT-549 (*n* = 6, *p* < 0.0001), and (**C**) Hs-578T (*n* = 4, *p* = 0.0001). All error bars shown represent standard error of the mean. Asterisks denote significance between control and each condition, whereas crosses indicate significance between shown conditions. *p*-value is for both asterisks and crosses. ** *p* < 0.01; *** *p* < 0.001; **** *p* < 0.0001; † *p* < 0.05; †† *p* < 0.01; †††† *p* < 0.0001.

We next sought to determine whether preventing the induction of SLFN12 using AdvshSLFN12 would prevent the effects of IFN-α2 on TNBC viability. In all three cell lines, IFN-α2 significantly reduced TNBC cell viability (Figure 2). However, a further reduction in cell viability was observed across each cell line when AdvShSLFN12 and IFN-α2 were combined, inconsistent with our original hypothesis that IFN-α2 would reduce cell viability by increasing SLFN2. 

### 3.3. SLFN Family mRNA Expression Variably Increases with Loss of SLFN12 and IFN-𝛼2 Signaling

**Figure 3:**
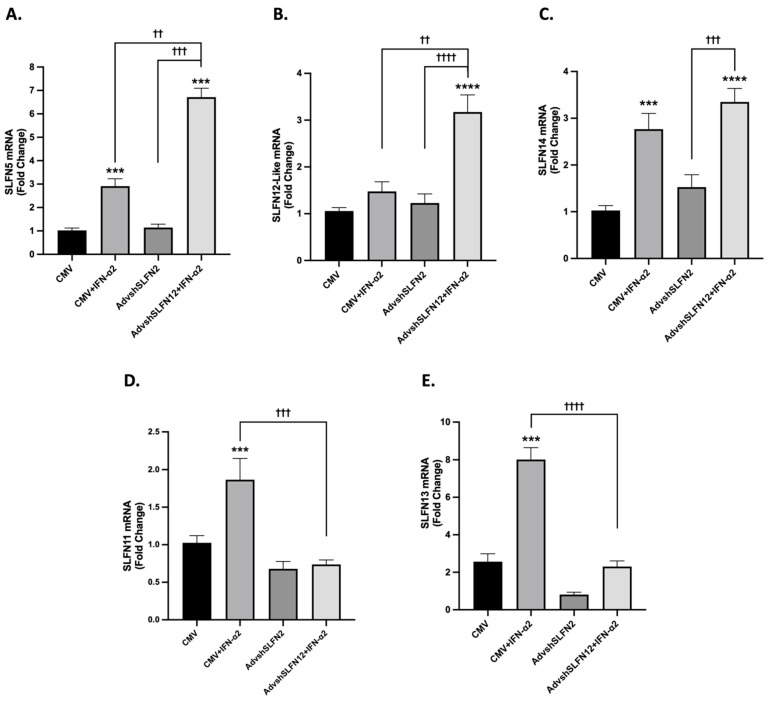
SLFN family expression variably increases with loss of SLFN12 and IFN-𝛼2 signaling. mRNA analysis by primer-probe RT-qPCR showed that (**A**) SLFN5 (*n* = 6, *p* < 0.0001), (**B**) SLFN12-Like (*n* = 6, *p* < 0.0001), and (**C**) SLFN14 (*n* = 6, *p* < 0.0001) are induced by IFN-𝛼2 treatment and significantly further induced with the loss of SLFN12 in MDA-MB-231 cells. This relationship indicates further SLFN family interplay in expression regulation. Contrariwise, (**D**) SLFN11 (*n* = 6, *p* = 0.0001) and (**E**) SLFN13 (*n* = 6, *p* < 0.0001) increased in expression due to IFN-𝛼2 treatment, but this increase was lost with the knockdown of SLFN12 in MDA-MB-231 cells, indicating that SLFN12 may regulate SLFN11 and 13 expression during IFN-𝛼2 signaling. RPLP0 is used as reference gene. All error bars shown represent standard error of the mean. Asterisks denote significance between control and each condition, whereas crosses indicate significance between shown conditions. *p*-value is for both asterisks and crosses. *** *p* < 0.001; **** *p* < 0.0001; †† *p* < 0.01; ††† *p* < 0.001; †††† *p* < 0.0001.

Since IFN-α2 reduced TNBC viability even when the induction of SLFN12 was prevented, we hypothesized that other SLFN family members might increase as a compensatory response to the loss of SLFN12 expression in this setting. To test this hypothesis, we analyzed the expression of SLFN family members in MDA-MB-231 cells after treatment with IFN-α2, AdvShSLFN12, and a combination of AdvShSLFN12 and IFN-α2. SLFN5 (Figure 3A), SLFN12-Like (Figure 3B), and SLFN14 (Figure 3C) were each also induced by IFN-α treatment alone and even further induced with the reduction in SLFN12. This relationship could suggest interaction among the SLFN family in controlling SLFN regulation. Conversely, SLFN11 (Figure 3D) and SLFN13 (Figure 3E) increased in expression due to IFN-α2 treatment but remained at control levels with SLFN12 knockdown, suggesting that SLFN12 may influence SLFN11 and SLFN13 expression in the setting of IFN-α2 signaling. Schlafen family expression in all three TNBC cell lines is summarized in Table 1. MDA-MB-231 expression with SLFN family members is exhibited in Figure 3, while SLFN family expression for BT-549 and Hs-578T cell lines are presented in Supplemental Figures S4 and S5, respectively.

### 3.4. SLFN11 and SLFN13 mRNA and Protein Expression Increase with IFN-𝛼2 Signaling

**Figure 4:**
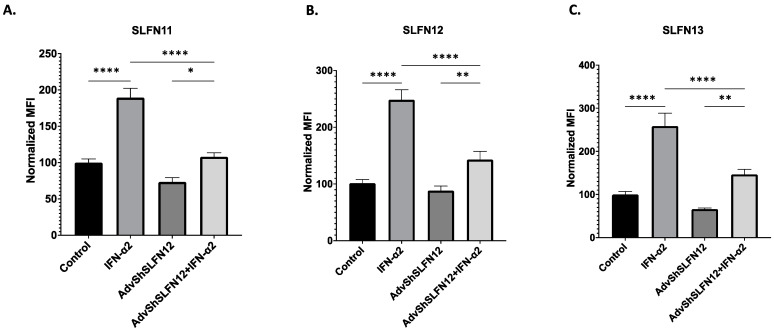
SLFN11 and SLFN13 mRNA and protein expression increase with IFN-α2 signaling. MDA-MB-231 protein levels were analyzed by flow cytometry to measure (**A**) SLFN11 (*n* = 16, *p* < 0.0001), (**B**) SLFN12 (*n* = 16, *p* < 0.0001), and (**C**) SLFN13 (*n* = 14, *p* < 0.0001). All 3 SLFNs showed an increase in protein expression following IFN-α2 treatment alone compared to control and the combination of AdvShSLFN12 with IFN-α2 in comparison to AdvShSLFN12 alone. There was no significant change in protein expression with the loss of SLFN12 alone in SLFN11, SLFN12, or SLFN13. All error bars shown represent standard error of the mean. * *p* < 0.05; ** *p* < 0.01; **** *p* < 0.0001.

To further evaluate the potential regulatory relationship between SLFN11, SLFN12, and SLFN13, we analyzed MDA-MB-231 SLFN protein levels via flow cytometry. The mRNA expression of SLFN11 and SLFN13 increased when the cells were stimulated with IFN-α2 (Figure 3D,E, Bar 2), but the treatment of AdvShSLFN12 and IFN-α2 prevented an increase in SLFN11 and SLFN13 levels (Figure 3D,E, Bar 4). The treatment with IFN-α2 yielded the highest protein expression in both SLFN11 and SLFN13 (Figure 4A,C, Bar 2), whereas the combination of AdvShSLFN12 and IFN-α2 did not solicit an increase in protein expression (Figure 4A,C, Bar 4). These protein results confirm the observed changes seen at the mRNA levels with SLFN11 and SLFN13. SLFN12 protein levels increased when treated with IFN-α2 but remained at a control level when treated with AdvShSLFN12 (Figure 4B, Bars 2–3). When IFN-α2 and AdvShSLFN12 were combined, we observed an increase in SLFN12 protein levels compared to AdvShSLFN12 alone (Figure 4B, Bar 4).

### 3.5. Apparent SLFN Interplay with IFN-α2 Signaling

**Figure 5:**
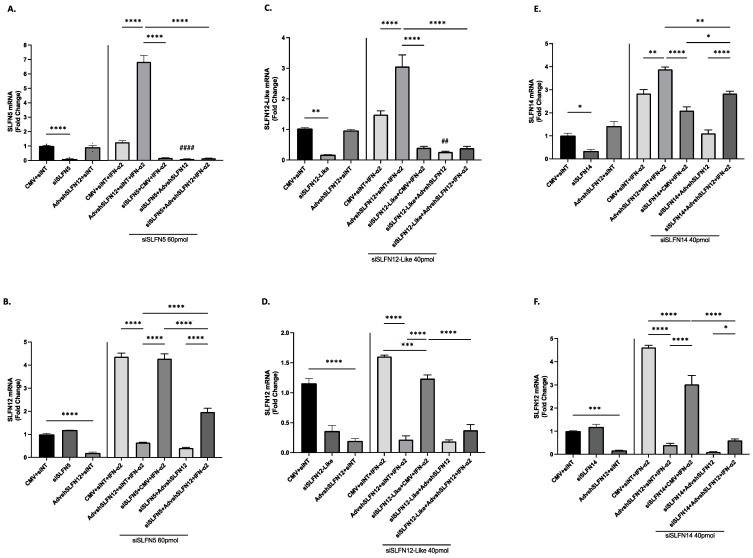
Apparent SLFN Interplay with IFN-α2 Signaling. mRNA analysis by primer-probe RT-qPCR in MDA-MB-231 cells evaluating the expression of (**A**) SLFN5 (*n* = 6, *p* < 0.0001), (**C**) SLFN12-Like (*n* = 6, *p* < 0.0001), (**E**) SLFN14 (*n* = 3, *p* < 0.0001), and (**B**,**D**,**F**) SLFN12 after respective siRNA treatments. Astricts denote significance between shown bars, and # denotes significance between AdvShSLFN12 + siNT and siSLFN5 + AdvShSLFN12 expression. *p*-value is for both asterisks and number signs. RPLP0 is used as reference gene. All error bars shown represent standard error of the mean. * *p* < 0.05; ** *p* < 0.01; *** *p* < 0.001; **** *p* < 0.0001; ## *p* < 0.01; #### *p* < 0.0001.

Unfortunately, it was not possible to design a single siRNA to interfere with the expression of all the SLFN family members because they lack a common sequence that can be targeted by this method; therefore, we used individually targeted siRNAs (Supplemental Tables S3 and S4) [17,18]. We used siRNA targeting SLFN5, SLFN12-Like, and SLFN14 to further examine the relationship of the SLFN family members that were synergistically induced by IFN-α2 and AdvShSLFN12 in TNBC. In MDA-MB-231 cells, siSLFN5 (Figure 5A), siSLFN12-Like (Figure 5C), and siSLFN14 (Figure 5E) were preliminarily analyzed to confirm successful siRNA knockdown of these SLFN proteins and then further examined with regard to their relationship to SLFN12. In the absence of SLFN5 or SLFN12-Like, there was no induction of SLFN5 or SLFN12-Like in the presence of IFN-α2, AdvShSLFN12, or the combination (Figure 5A,C, Bars 6–8). Alternatively, when SLFN12 was absent, SLFN14 expression was induced in the presence of IFN-α2 (Figure 5E, Bars 4–5). SLFN14 was not induced in the absence of SLFN12 and SLFN14, but when IFN-α2 and AdvShSLFN12 were combined, we once again saw an induction in SLFN14 mRNA levels (Figure 5E, Bars 6–8).

When SLFN5 was knocked down with siSLFN5, the IFN-α2 induction of SLFN12 expression was not lost, suggesting that the loss of SLFN5 does not influence SLFN12 expression (Figure 5B Bar 6). When SLFN12-Like was knocked down, we observed a loss in SLFN12 expression (Figure 5D Bar 2). Therefore, SLFN12 expression appears to be partially reliant on SLFN12-Like during IFN-α2 signaling because we detected a partial decrease in SLFN12 expression, but not to the control level (Figure 5D Bar 6). When we knocked down SLFN14, there was no effect on SLFN12 in the control state (Figure 5F Bar 2). When SLFN14 was lost during IFN-α2 treatment, SLFN12 expression decreased, but not to the control level (Figure 5F Bar 6).

### 3.6. IFN-𝛼2 Decreases TNBC Cell Viability with Simultaneous Knockdown of SLFN5, SLFN12, SLFN12-Like, and SLFN14

**Figure 6:**
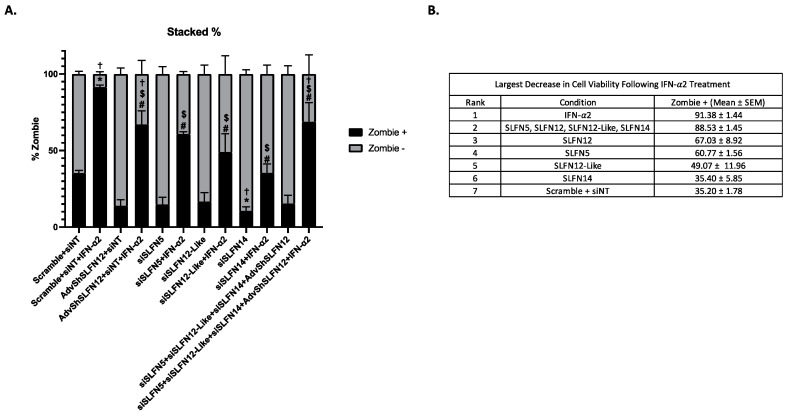
IFN-α2 decreases TNBC cell viability with simultaneous knockdown of SLFN5, SLFN12, SLFN12-Like, and SLFN14. MDA-MB-231 cells were analyzed by flow cytometry with Zombie Aqua™ Fixable Viability Kit. (**A**) SLFN5, SLFN12, SLFN12-Like, and SLFN14 (*n* = 6, *p* < 0.0001) were individually knocked down, treated with IFN-α2 individually and in combination. Live cells are displayed as Zombie, and dead cells are represented by Zombie +. Two-way ANOVA analysis was performed. * denote significance to Scramble + siNT, † specifies significance to Scramble + siNT; # shows the comparison of Scramble + siNT + IFN-α2 vs. AdvshSlfn12/siRNAs + IFN-α2, and $ indicates the comparison of each AdvshSlfn12/siRNA vs. its respective AdvshSlfn12/siRNA + IFN-α2. All error bars shown represent standard error of the mean. (**B**) Treatments were then ranked by the largest decrease in cell viability following IFN-α2 treatment by the mean and SEM of the Zombie + cell values. *, †, #, $ *p* < 0.05.

To evaluate whether the Schlafen proteins may act together, we simultaneously knocked down SLFN5, SLFN12, SLFN12-Like, and/or SLFN14 to determine whether this would negate the effect of IFN-α2 on cell viability. TNBC cell viability was assessed via flow cytometry to determine the percent of live (Zombie −) and dead cells (Zombie +) (Figure 6A). IFN-α2 yielded the largest decrease in cell viability (Figure 6A, Zombie +, Bar 2). Individual knockdown of SLFN12, SLFN5, or SLFN12-Like with IFN-α2 led to partial but substantial attenuation of the IFN-α2 effect on decreased cell viability, while siSLFN14 + IFN-α2 treatment restored cell viability to similar levels, as seen in the Scramble + siNT control. Additionally, treatment with the siRNA or adenovirus hairpin to SLFN family members alone resulted in partial yet substantial improvement in cell viability compared to the Scramble + siNT control. When all 4 SLFNs were knocked down and IFN-α2 was added (Bar 12), we observed a modest but statistically significant improvement in cell viability in comparison to the cells treated with Scramble + siNT + IFN-α2 but not nearly as great an improvement in cell viability as when each of the SLFN family members was individually knocked down. A full summary of these effects on cell viability, represented by the mean percent ± SEM of Zombie + cells, is presented in Figure 6B.

## 4. Discussion

Exogenously increasing SLFN12 reduces the cell viability of TNBC cells. In this study, we observed that knockdown in SLFN5, SLFN12, or SLFN12-Like proteins partially recovered cell viability following IFN-α2 signaling, whereas SLFN14 knockdown led to a full recovery of cell viability. SLFN5, SLFN12-Like, and SLFN14 are not controlled by SLFN12 during IFN-α2 signaling, whereas SLFN12 may be necessary for SLFN11 and SLFN13 induction during IFN-α2 signaling. Therefore, these data suggest that SLFN family members may indeed contribute to the IFN-α2 mitogenic effect but in a complex fashion that may reflect both the interplay among pro-survival and anti-survival effects of some SLFN family members and the interactions by which one SLFN family member influences the expression of others [3,7,12].

The effects of IFN-α2 on cell viability appear to be mediated at least in part in a complex fashion through changes in the expression of the SLFN proteins. We originally sought an extracellular pharmacologic treatment that would increase SLFN12 expression. IFN-α2 stimulates SLFN gene expression, which, in turn, regulates cell cycle progression, collagen invasion, and matrix re-organization [19,20,21,22]. In melanoma cells, IFN-α2 is able to induce only human SLFN5 gene expression, while other SLFN family members were not induced [23]. In our studies of TNBC cell lines, IFN-α2 increased the expression of all SLFN family members. The inductions of SLFN11 and SLFN13 in TNBC by IFN-α2 required SLFN12, while IFN-α2 induction of SLFN5, SLFN12-Like, or SLFN14 mRNA expressions did not. These results cohere with previous work suggesting that loss of the mouse SLFN12 ortholog, Slfn3, can affect the expression of the other SLFN members. In mice, the loss of Slfn3 changes the expression of other murine Slfn family members in the intestinal mucosa, thymus, and spleen in a complex fashion [17]. The loss of Slfn3 also influences the expression of other Schlafen family members after 50% bowel resection in mice in a sex-dependent manner [17,18]. 

Contrary to our original hypothesis, the effects of IFN-α2 on TNBC cells do not appear to be purely mediated through SLFN12. To explore our observation that cell viability was unexpectantly still decreased when SLFN12 was knocked down during IFN-α2 treatment, but other SLFN family members were increased, we first reduced each SLFN protein in turn. While SLFN5, SLFN12, or SLFN12-Like knockdown during IFN-α2 treatment resulted in only a partial cell viability recovery, SLFN14 knockdown completely blocked the effects of IFN-α2 on cell viability. Publicly available database analysis reveals that SLFN14 is downregulated in breast cancer and has a positive survival correlation to this malignancy [6]. SLFN14 is a ribosome-associated nuclear protein that binds to the ribosomal subunits and cleaves RNA, in particular rRNA and ribosome-associated mRNA, to control mRNA turnover and protein translation [6,24,25]. Additionally, IFN-I upregulates the expression of SLFN14 in various benign and malignant neural cells, indicating that SLFN14 may be a viable target moving forward [6,24,25].

Indeed, members of the SLFN family influence each other’s expression in the setting of IFN-α2 stimulation in a complex fashion, but there are also some restrictions to this study. Unfortunately, it was not possible to design a single siRNA to interfere with the expression of all the SLFN family members because they lack a common sequence that can be targeted by this method [26,27]. The use of a single siRNA would decrease the likelihood of the possible induction of off-target effects while also providing enhanced siRNA delivery, stronger gene silencing, and more potent anticancer activity compared to utilizing individual SLFN siRNA molecules [28,29]. It seems paradoxical that preventing the effect of IFN-α2 on SLFN14 blocked the IFN-𝛼2 effect on cell viability, but reducing all the SLFN proteins only resulted in a partial recovery [27,28]. Conversely, the SLFN family members may function as an intra-regulated rheostat with some redundancy in expressions and function since the SLFN paralogues are clustered together and have some structural similarity [17,30,31,32]. The SLFN family members may require a balanced expression to maintain or decrease cell viability. Additionally, IFN-α serum levels fluctuate in different pathological conditions. IFN-α activity in normal healthy serum is <2 IU/mL [33,34] compared to 430 IU/mL in individuals with systematic lupus erythematosus (SLE) [35]. In our in vitro setting, we used 5500 IU/mL of IFN-α, whereas, in in vivo, IFN-α is administered at 9 million U subcutaneously up to three times a week in cancer therapy [36,37]. In the future, therapeutic target design would need to target multiple SLFN family members carefully since they regulate each other during IFN-α2 signaling. Additional studies will need to be completed before these techniques can be designed.

**Figure 7:**
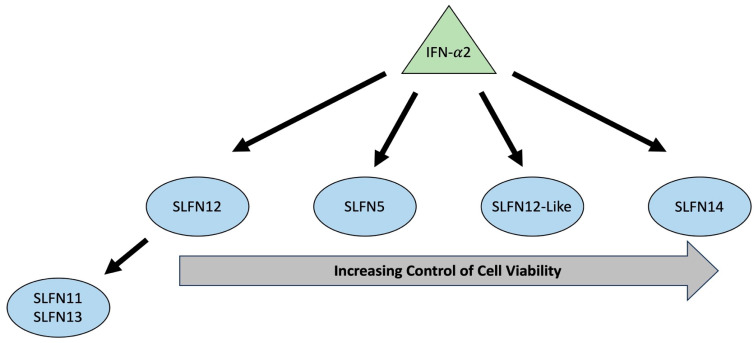
Proposed IFN-α2 signaling cascade. IFN-α2 signaling cascade that illustrates a novel intra-regulation between Schlafen family members and increasing control of cell viability.

Based on our observations, we propose a complex interaction among the SLFN family by which various SLFNs influence each other’s expression during IFN-α2 signaling (Figure 7). In addition, our results suggest a cascade of how Schlafen family members influence cell viability. SLFN11 and SLFN13 are dependent on SLFN12 during IFN-α2 signaling for mRNA expression and protein regulation. Schlafen family members control cell viability following IFN-α2 treatment, with increasing control going from SLFN12, SLFN5, SLFN12-Like, to SLFN14.

## 5. Conclusions

IFN-α2 treatment induces SLFN5, SLFN11, SLFN12, SLFN12-Like, SLFN13, and SLFN14 expressions in TNBC while simultaneously reducing cell viability. Following SLFN12 knockdown during IFN-α2 signaling, cell viability was still reduced, indicating that IFN-α2 signaling is not exclusively acting through SLFN12. Further exploration into SLFN family member expression indicated that SLFN5, SLFN12-Like, and SLFN14 are not controlled by SLFN12 during IFN-α2 signaling. Conversely, SLFN12 may be necessary for SLFN11 and SLFN13 induction during IFN-α2 signaling. These results may indicate an ordered control of cell viability during IFN-α2 signaling by SLFN family members, where the control is highest with SLFN14 > SLFN12-Like > SLFN5 > SLFN12. But with only a slight improvement in cell viability when all the SLFN members are knocked down, it could indicate an intra-regulating role for each SLFN family member in controlling cell viability during IFN-α2 signaling. Overall, these data indicate a complex and novel intra-regulated signaling cascade between SLFN family members, which may provide insight into delivering targeted therapy for TNBC patients.

## Figures and Tables

**Table 1:** SLFN Family qPCR Fold Change Analysis.

	IFN-α vs. AdvShSLFN12 + IFN-α			AdvShSLFN12 vs. AdvShSLFN12 + IFN-α		
	MDA-MB-231	BT549	Hs-578T	MDA-MB-231	BT549	Hs-578T
SLFN5	↑↑	↑↑	↑	↑↑↑	↑	↓↓
SLFN11	↓	↓↓↓	↑	-	-	-
SLFN12	↓↓	↓↓	↓	-	-	↑
SLFN12-like	↑	↑	↑↑	↑↑	↑	-
SLFN13	↓↓↓	↑	↑	↑	-	-
SLFN14	-	↑	↑↑	↑	↑	↑↑

↑ Increase in mRNA Expression; - No Change in mRNA Expression; ↓ Decrease in mRNA Expression.

## Data Availability

Supporting data from this study will be made available upon request.

## Supplementary Materials

The following supporting information can be downloaded at https://www.mdpi.com/article/10.3390/cancers15235658/s1, Figure S1: MDA-MB-231 qPCR with IFN-α2; Figure S2: BT-549 qPCR with IFN-α2; Figure S3: Hs-578T qPCR with IFN-α2; Figure S4: SLFN family expression variably increases with loss of SLFN12 and IFN-α2 Signaling in BT-549 Cells; Figure S5: SLFN family expression variably increases with loss of SLFN12 and IFN-α2 Signaling in Hs-578T Cells; Table S1: Primers; Table S2: Antibodies; Table S3: siRNA Information; Table S4: siRNA Percent Reduction.
