# Supplementary material for: Schlafen Family Intra-Regulation by IFN-α2 in Triple-Negative Breast Cancer
Source: Cancers (Basel). 2023 Nov 30;15(23):5658. doi: 10.3390/cancers15235658 (PMC10705374; doi:10.3390/cancers15235658)
Supplement: Supplementary file 1 [file cancers-15-05658-s001.zip › Supp Table S1.pdf]

Table 1. qPCR primers

Gene	Forward	Reverse	Probe
Human RPLPO	5’-GCAATGTTGCCAGTGTCTG-3’	5’-GCCTTGA CC TTT TCAGCAA-3’	
Human SLFN5	5'-TGGCAGATGACAAACTCAACT-3'	5'-GAGAATGGACTGCTTGGATGA-3'	5'-/56-FAM/CCTGGAAAG/ZEN/GTCTGGGTCAGCTT/3IABkFQ/-3'
Human SLFN11	5'-GCTGGAGTTCCTTTTTATGTTCC-3'	5'-GCATGATGACAGACACAGATCC-3'	5'-/56-FAM/CTTCCCCTT/ZEN/AGCAGACCAGTGTACT/3IABkFQ/-3'
Human SLFN12	5’- GGGAGCAGGTAATGACGTATTTATT- 3’	5’- CAGTTGACCAGGAAGGAATGG- 3’	5’-/56-FAM/ATCCAGTTC/ZEN/ ATGGTGGAGGCTGAA/3IABkFQ/–3’
Human SLFN12-Like	5'-GCTCAGCATAGTTTGTGTCTAA-3'	5'-ATGGACCTCGCCAGAAA-3'	5'-/56-FAM/TGGAAATGG/ZEN/CTTAGCTGCTGGGAA/3IABKFQ/-3'
Human SLFN13	qHsaCEP0052911		
Human SLFN14	qHsaCEP0056002		
