# Supplementary material for: Schlafen Family Intra-Regulation by IFN-α2 in Triple-Negative Breast Cancer
Source: Cancers (Basel). 2023 Nov 30;15(23):5658. doi: 10.3390/cancers15235658 (PMC10705374; doi:10.3390/cancers15235658)
Supplement: Supplementary file 1 [file cancers-15-05658-s001.zip › Supp Table S2.pdf]

Table 2. Antibody Information				
**Antibody**	**Host Species**	**Dilution**	**Company**	**Catalog Number**
Anti-SLFN11 PE	Rabbit	1:50	Cell Signaling Technology	34858
Anti-SLFN12 APC	Rabbit	1:25	LSBio	LS-B4757-0.05
Anti-SLFN13	Rabbit	1:50	Novus Biologicals	NBP1-93879
Anti-Rabbit IgG Secondary	Mouse	1:100	Novus Biologicals	NBP2-50261AF647
