# Supplementary material for: Schlafen Family Intra-Regulation by IFN-α2 in Triple-Negative Breast Cancer
Source: Cancers (Basel). 2023 Nov 30;15(23):5658. doi: 10.3390/cancers15235658 (PMC10705374; doi:10.3390/cancers15235658)
Supplement: Supplementary file 1 [file cancers-15-05658-s001.zip › Supp Table S3.pdf]

Table 3. siRNA Information

	Mixture A	
	**Opti-MEM**	**Lipo RNAiMAX**
	150 μL	16.5 μL

	Mixture B	
	**Opti-MEM**	**siRNA**
**siNT [40 pmol]**	150 μL	9 μL
**siSLFN5 [60 pmol]**	150 μL	6 μL
**siSLFN12-Like [40 pmol]**	150 μL	4 μL
**siSLFN14 [40 pmol]**	150 μL	4 μL
