# Supplementary material for: Schlafen Family Intra-Regulation by IFN-α2 in Triple-Negative Breast Cancer
Source: Cancers (Basel). 2023 Nov 30;15(23):5658. doi: 10.3390/cancers15235658 (PMC10705374; doi:10.3390/cancers15235658)
Supplement: Supplementary file 1 [file cancers-15-05658-s001.zip › Supp Table S4.pdf]

Table 4. siRNA Percent Reduction

	40 pmol	60 pmol
**siSLFN5**	40.47	**89.06**
**siSLFN12-Like**	**83.00**	76.47
**siSLFN14**	**65.74**	31.45
