# Supplementary figures and images for: Schlafen Family Intra-Regulation by IFN-α2 in Triple-Negative Breast Cancer
Source: Cancers (Basel). 2023 Nov 30;15(23):5658. doi: 10.3390/cancers15235658 (PMC10705374; doi:10.3390/cancers15235658)

**A.**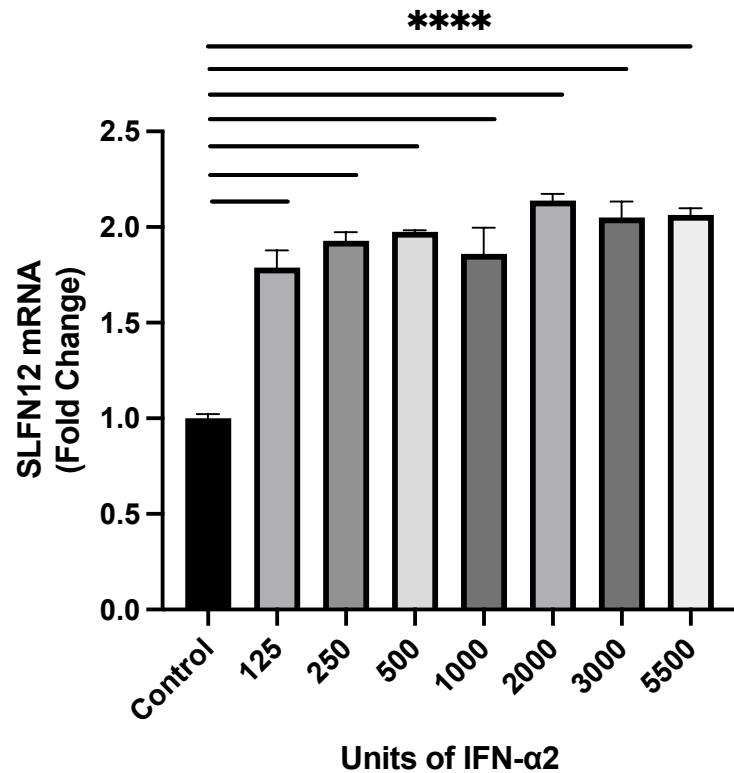**B.**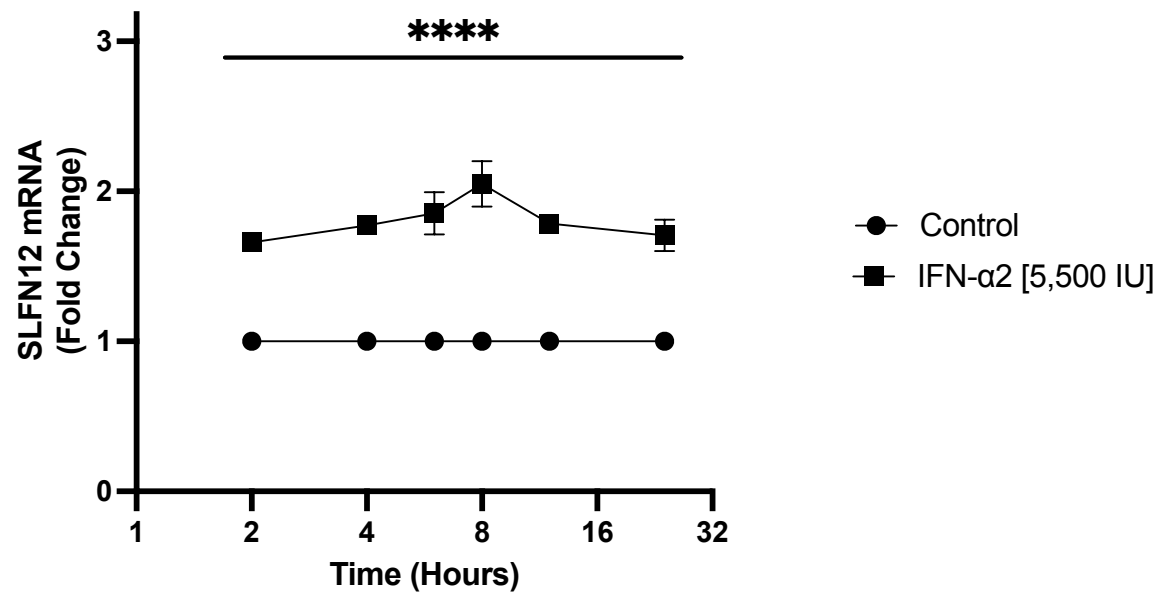

Supplement: Supplementary file 1 [file cancers-15-05658-s001.zip › Supp Figure S1.pdf]

**A.**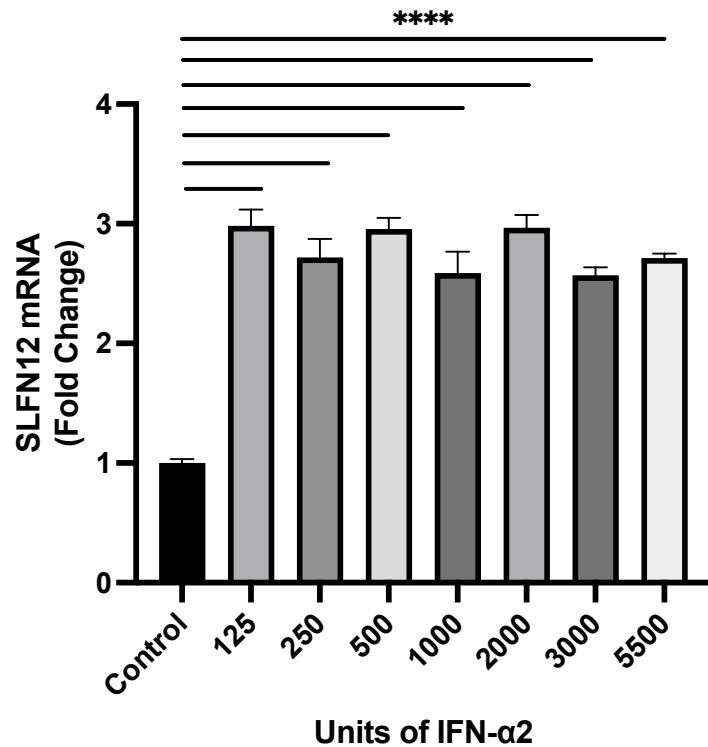**B.**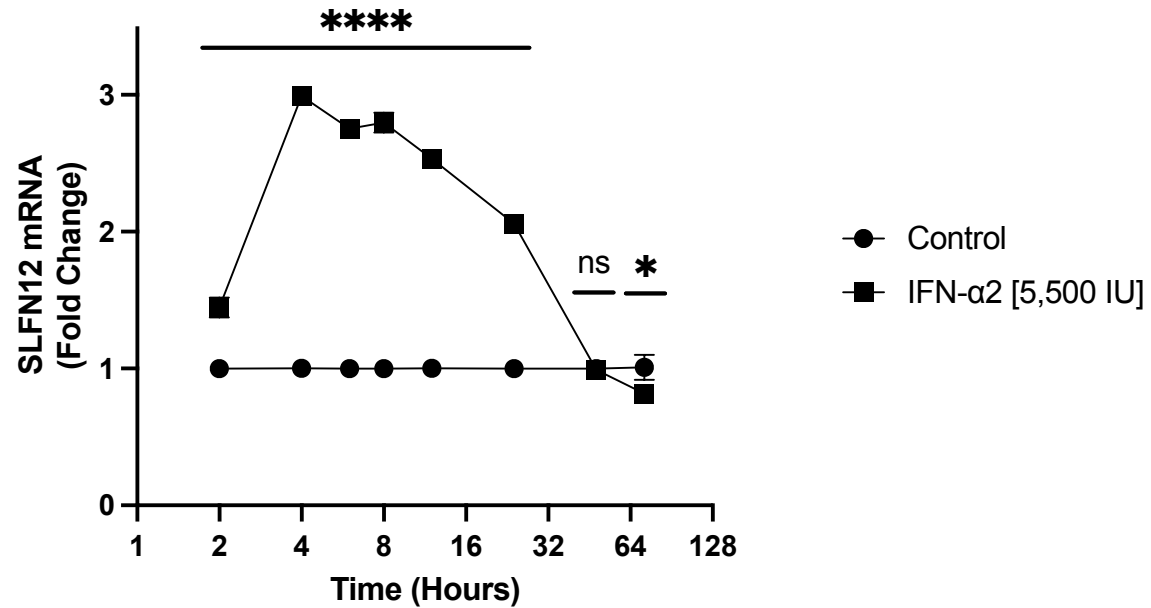

Supplement: Supplementary file 1 [file cancers-15-05658-s001.zip › Supp Figure S2.pdf]

**A.**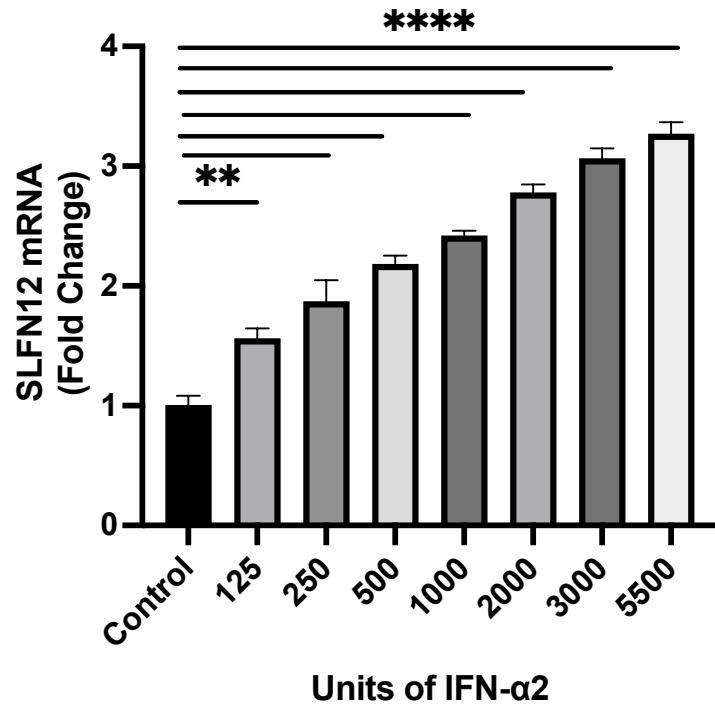**B.**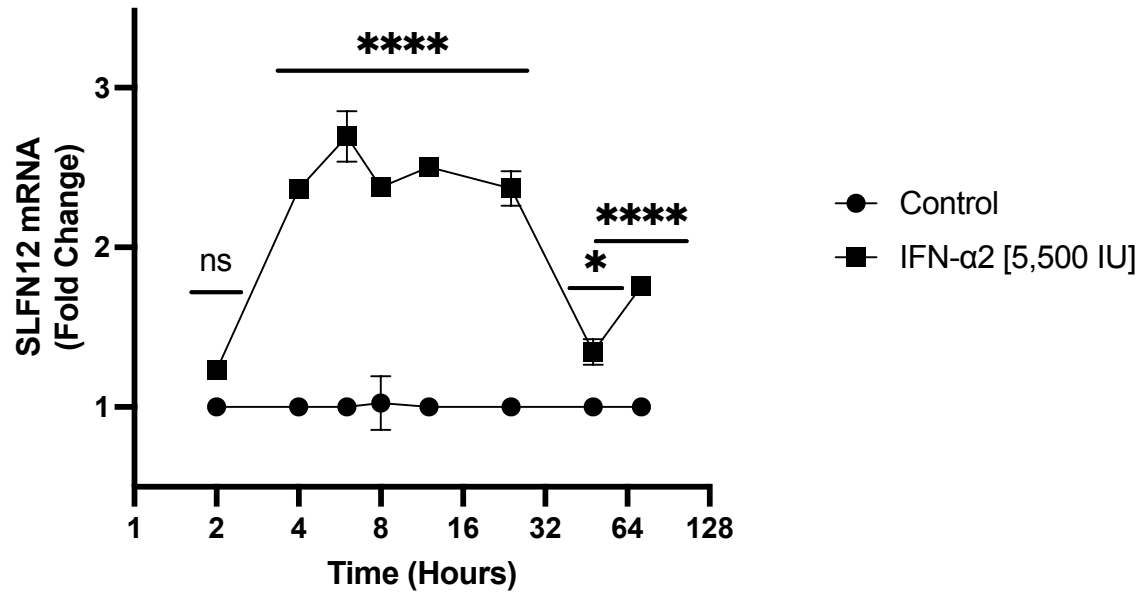

Supplement: Supplementary file 1 [file cancers-15-05658-s001.zip › Supp Figure S3.pdf]

A.

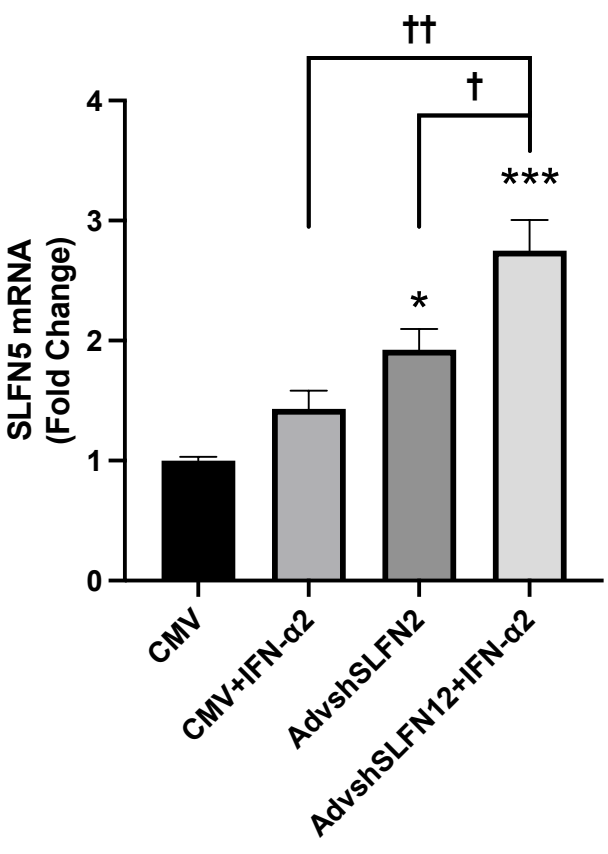

B.

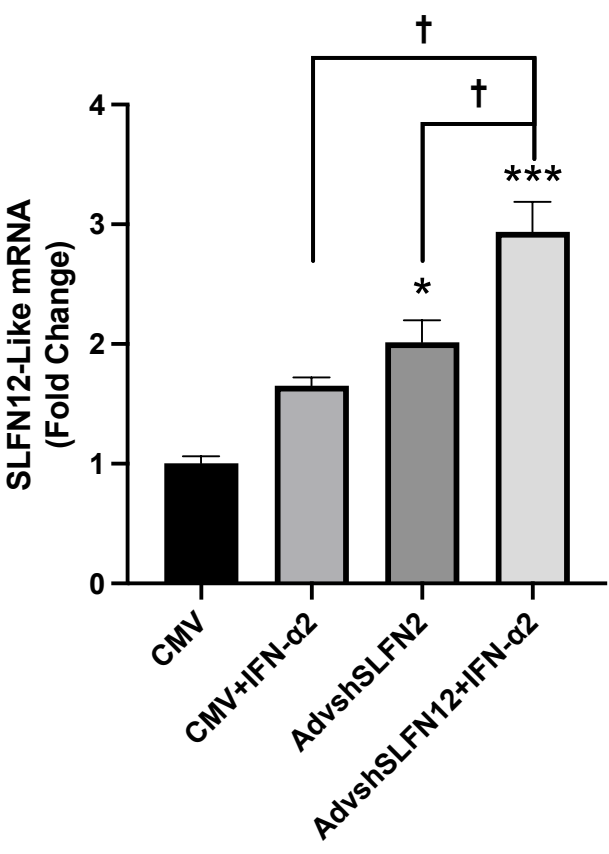

C.

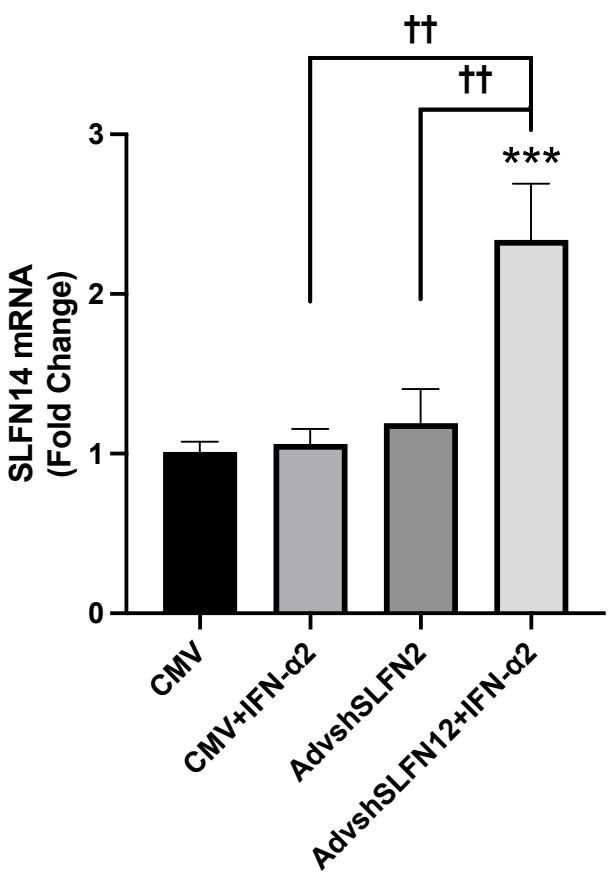

D.

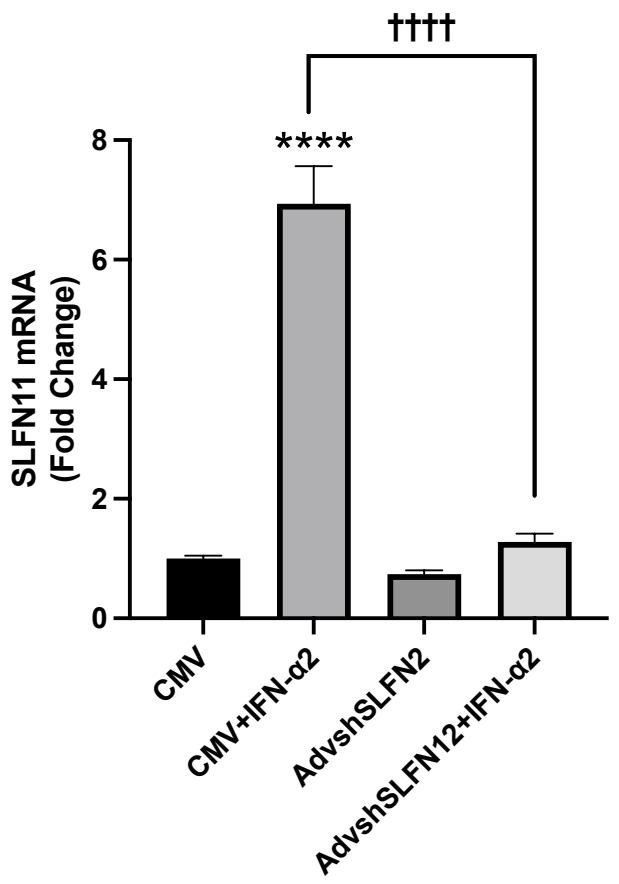

E.

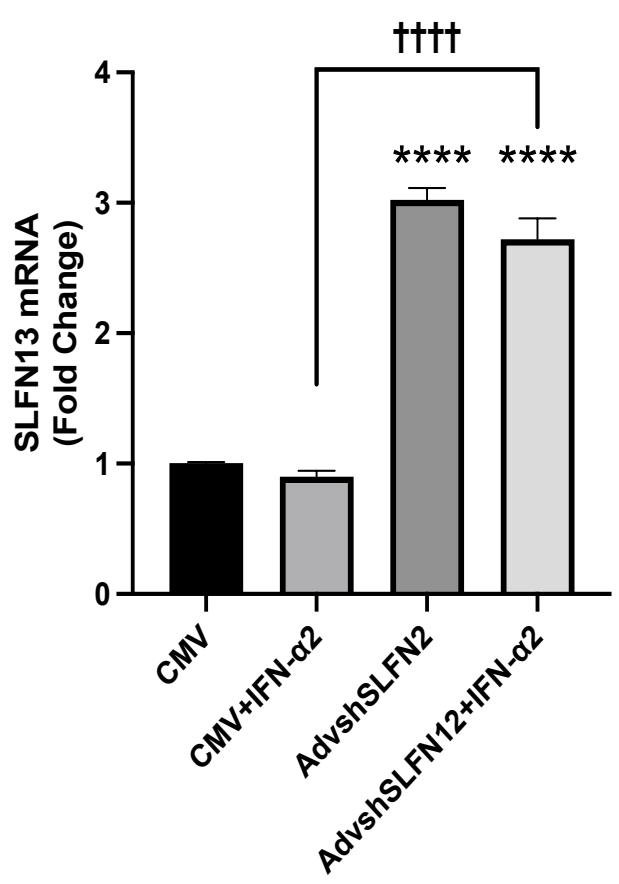

Supplement: Supplementary file 1 [file cancers-15-05658-s001.zip › Supp Figure S4.pdf]

**A.**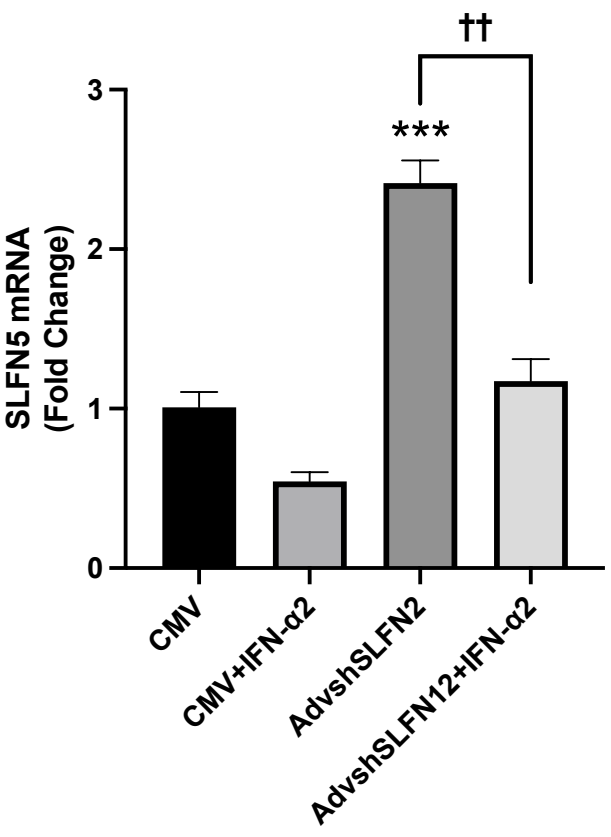**B.**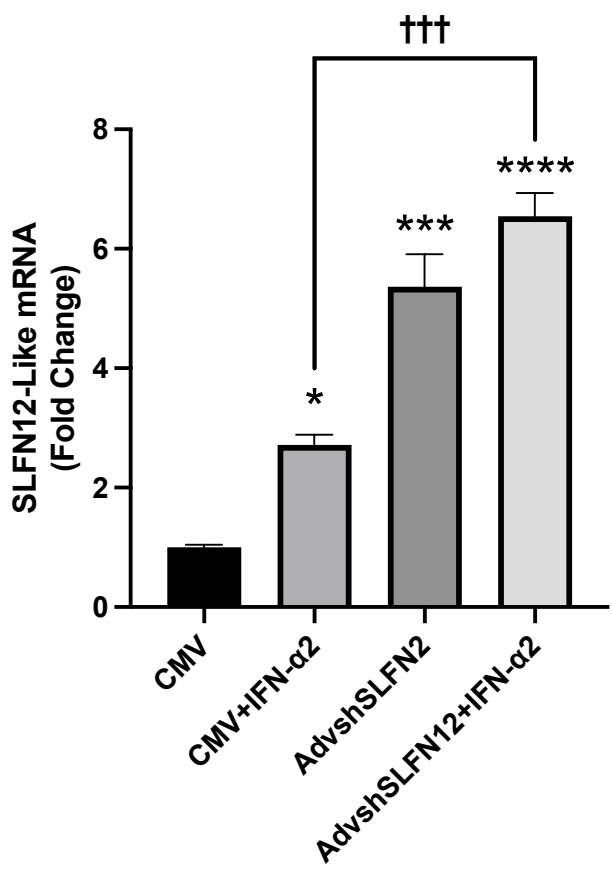**C.**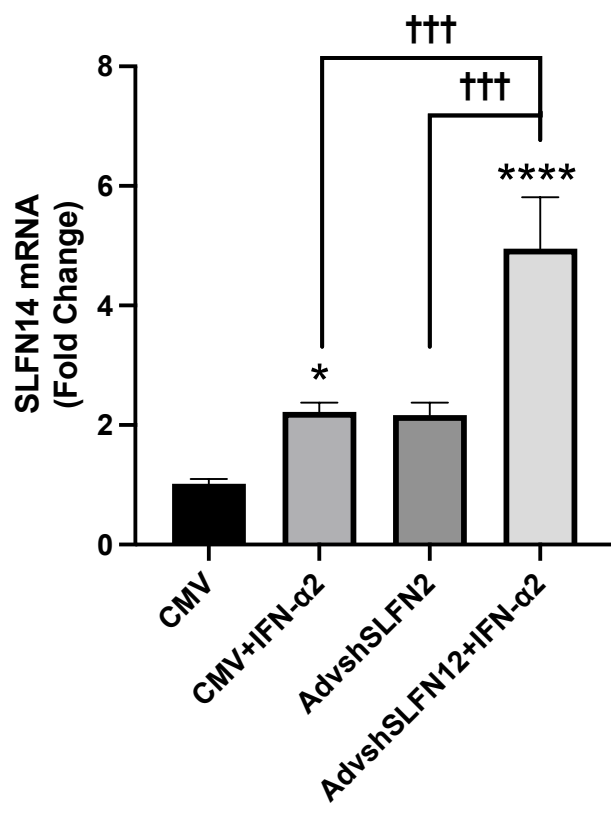**D.**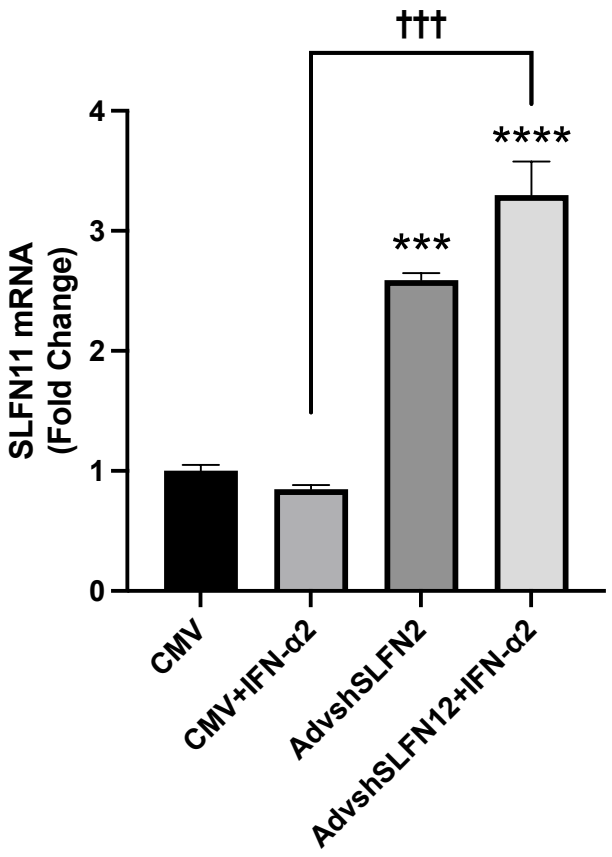**E.**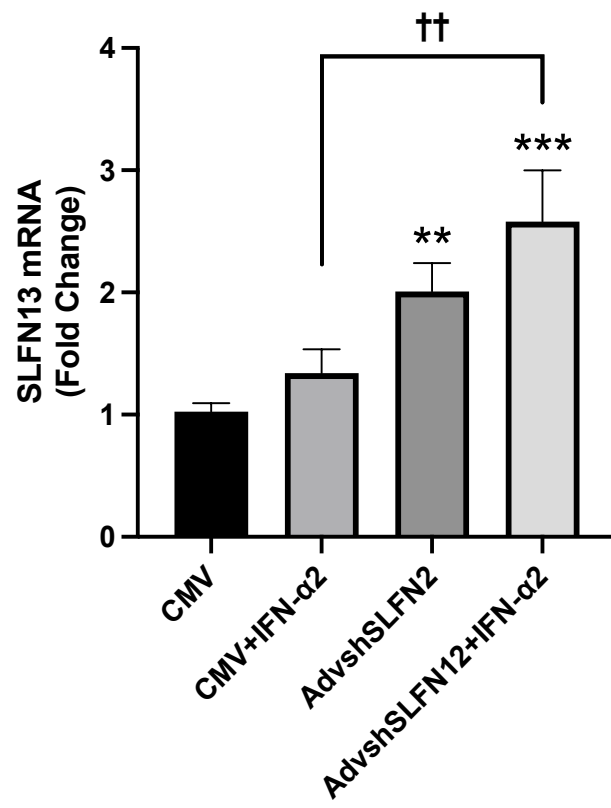

Supplement: Supplementary file 1 [file cancers-15-05658-s001.zip › Supp Figure S5.pdf]
